# A Video Interview With Louis Strock, MD

**DOI:** 10.1093/asjof/ojab054

**Published:** 2021-12-13

**Authors:** Nicole R Vingan, Louis Strock, Jeffrey M Kenkel

**Affiliations:** Department of Plastic Surgery, University of Texas Southwestern Medical Center, Dallas, TX, USA

*Second Thoughts on First Thoughts* is a thought-provoking section intended for experienced plastic surgeons to discuss and reflect upon changes they have made to their craft over time. As knowledge, technology, and ability advances, it is also important for surgeons to evolve and refine their approaches.^1^ The purpose of this elucidating segment is to ultimately provide colleagues with pearls of knowledge gleaned from surgeons who have evolved their practices over time. In this article, we will explore the evolution of Dr. Louis Strock's, approach to achieving breast height in his surgical patients. 

## WHAT I USED TO DO

Throughout his career, Dr Strock has emphasized the goal of ensuring that his patients leave the operating room with adequate breast height following breast surgery. During his years in practice, Dr Strock has spent a lot of time preoperatively assessing the proper choice of implants that will compensate for lack of existing height (Video). The use of teardrop-shaped, highly cohesive gel implants proved to be a reliable choice. This choice of implant can provide patients with a form stable structure that is taller than it is wide, which tends to minimize some of the clinically significant rotation issues that can occur with alloplastic breast reconstruction.

## WHY I MADE A CHANGE AND HOW I DO IT NOW

Dr Strock’s second thoughts on his approach to achieving breast height were prompted in 2017 following the development of newer devices, such as the Allergan Soft Touch implant (Irvine, CA) and Mentor Memory Gel Xtra implant (Irvine, CA) product lines. He chose to reinvent his approach, specifically patients undergoing breast implant revision and augmentation mastopexy, to predictably achieve the higher footprint and upper pole breast fullness he desires for all of his patients. Dr Strock now utilizes these newer iterations of round smooth wall gel implants that are either more cohesive or more highly filled, to try and create height, as well as an absorbable internal soft tissue support via poly-4-hydroxybutyrate (P4HB) scaffolding. The more highly filled devices can provide a tighter feel with varying degrees of softness, while the highly cohesive implants can retain shape more uniformly. Additionally, the P4HB scaffold can provide the necessary soft tissue stability to reduce unwanted implant movement. 

## SUMMARY

Dr Strock is well known for his expertise in implant-based aesthetic and reconstructive breast surgery. The extensive experience and knowledge he has garnered through his years in practice have allowed him to consistently provide his patients with their personalized breast implant needs. This attention to detail and diligent pursuit for excellence embodies the crux of this *ASJ Open Forum* feature. Dr Strock’s commitment to his patients and to advancing his practice of alloplastic breast reconstruction prompted him to have second thoughts, reevaluate his initial surgical and implant approach, and improve his techniques for the future.

## References

[1] Rosenfield LK. On second thought. Aesthet Surg J Open Forum. 2020;2(1):1-2. doi: 10.1093/asjof/ojz033 PMC7671287 33791633

